# An Overview of the World Current and Future Assessment of Novel COVID-19 Trajectory, Impact, and Potential Preventive Strategies at Healthcare Settings

**DOI:** 10.3390/ijerph17197016

**Published:** 2020-09-25

**Authors:** Bader S. Al-Anzi, Mohammad Alenizi, Jehad Al Dallal, Frage Lhadi Abookleesh, Aman Ullah

**Affiliations:** 1 Department of Environmental Technology Management, Kuwait University, P.O. Box 5969, Safat 13060, Kuwait; 2 General Department of Criminal Evidences Identification, Ministry of Interior, Safat 12003, Kuwait; mohd_80@hotmail.com; 3 Department of Information Sciences, Kuwait University, P.O. Box 5969, Safat 13060, Kuwait; j.aldallal@ku.edu.kw; 4 Department of Agricultural, Food and Nutritional Science, University of Alberta, Edmonton, AB T6G 2P5, Canada; abooklee@ualberta.ca (F.L.A.); ullah2@ualberta.ca (A.U.)

**Keywords:** COVID-19, trajectory, exponential model, polynomial model, death percentage, containment strategies

## Abstract

This study is an overview of the current and future trajectory, as well as the impact of the novel Coronavirus (COVID-19) in the world and selected countries including the state of Kuwait. The selected countries were divided into two groups: Group A (China, Switzerland, and Ireland) and Group B (USA, Brazil, and India) based on their outbreak containment of this virus. Then, the actual data for each country were fitted to a regression model utilizing the excel solver software to assess the current and future trajectory of novel COVID-19 and its impact. In addition, the data were fitted using the Susceptible–Infected–Recovered (SIR) Model. The Group A trajectory showed an “S” shape trend that suited a logistic function with r^2^ > 0.97, which is an indication of the outbreak control. The SIR models for the countries in this group showed that they passed the expected 99% end of pandemic dates. Group B, however, exhibited a continuous increase of the total COVID-19 new cases, that best suited an exponential growth model with r^2^ > 0.97, which meant that the outbreak is still uncontrolled. The SIR models for the countries in this group showed that they are still relatively far away from reaching the expected 97% end of pandemic dates. The maximum death percentage varied from 3.3% (India) to 7.2% with USA recording the highest death percentage, which is virtually equal to the maximum death percentage of the world (7.3%). The power of the exponential model determines the severity of the country’s trajectory that ranged from 11 to 19 with the USA and Brazil having the highest values. The maximum impact of this COVID-19 pandemic occurred during the uncontrolled stage (2), which mainly depended on the deceptive stage (1). Further, some novel potential containment strategies are discussed. Results from both models showed that the Group A countries contained the outbreak, whereas the Group B countries still have not reached this stage yet. Early measures and containment strategies are imperative in suppressing the spread of COVID-19.

## 1. Introduction

Today’s world is changing rapidly at different levels during the current technological and medical renaissance due to Research and Development (R&D) that has resulted in innovative technologies and outcomes. Sometimes such developments may lead to disastrous consequences causing adverse effects on the environment, which could be reflected on the health of living organisms at different levels such as global warming as a part of air pollution, water pollution, and even cancer. Some of the advancements could be in the medical field, as well some new antibiotics and vaccines to fight new diseases and outbreaks. As a result, some microorganisms/pathogens have mutated to develop resistance to existing treatments causing many epidemics and outbreaks. 

Disease and sickness have tormented humanity since the beginning of life on earth. Human beings, along with plants and vegetables, have had to face challenges from microorganisms during their evolution. The mankind history has seen epidemics characterized by mortality and morbidity [1]. However, the scale of these diseases has increased notably since the advent of globalization. With the rise in global trade, expansion of civilization, contact with populations across the globe, new opportunities for human interactions have become more common, resulting in the proliferation of such epidemics. The early years witnessed the onset of several diseases such as Malaria, smallpox, tuberculosis, influenza, leprosy, whose cure has eventually been discovered [2]. 

The scientific efforts made during the mid-20th century has reduced the spread of epidemics in the world, mainly due to the advances in medical services, improvements in health care and the urban environment, and availability of vaccines and antibiotics [1,3]. However, the widespread outbreaks have again returned to the world in the 21st century, possibly due to pollution, overpopulation, global transportation network, and poverty in some parts of the world. Hence, the recurrence and emergence of potential infectious diseases that may cause epidemics is most likely to continue [3]. In fact, the World Health Organization (WHO) has claimed that approximately 15 million deaths due to these infectious diseases takes place each year. In developing countries with the least economic resources, such diseases are the major causes of deaths. Certain diseases such as tuberculosis and malaria, have reappeared due to the emergence of drug resistant microorganism strains. Lastly, the perception of “deliberately emergent” pathogens (such as anthrax and smallpox), and the possibility of their use for bioterrorism in the contemporary world cannot be ruled out [4].

Recently, the world has gone through an intermittent communicable disease outbreak that led to unprecedented epidemics, which have significantly impacted humanity claiming many innocent lives and the economy. Such epidemics are Sever Acute Respiratory Syndrome (SARS), Middle Eastern Respiratory Syndrome (MERS), and of course the current novel Coronavirus that has been classified as a pandemic (COVID-19). Table 1 lists the historic and recent epidemics/pandemics [3,5] that have occurred in the world. No one can overlook what the world is going through these days as a result of the COVID-19 pandemic that has already spread through the 213 countries affecting 13,000,000 people and, unfortunately, killing around 600,000 people over a short timeframe (5–7 months), still with an accelerated pace and notable upward trend [6]. As aforementioned, this has affected the world economy, the humans’ life, and has spread panic all around. On 31 December 2019, there was a cluster of pneumonia cases in Wuhan, Hubei province, China, whose investigation revealed that these cases were associated with the novel coronavirus or COVID-19, as it is called today [7]. 

Viruses are not from the plant or animal kingdom, and are neither bacteria, but are the typical parasites of the living kingdoms. Viruses are not living organisms because they cannot live without a host cell. All viruses contain a core, made of a genetic material—nucleic acid, either DNA or RNA—and a protein shell, which encases the nucleic acid [8]. Coronaviruses are a large group of viruses [9] surrounded by an envelope with protein spikes, which gives the appearance of a crown (or, in Latin, corona) from where it derives its name. [10]. There are different types of coronaviruses that cause respiratory and gastrointestinal problems [11]. Respiratory diseases can range from pneumonia and in most people the symptoms can generally cause a mild disease [12]. However, there are some types of coronaviruses that can cause several diseases such as SARS: The coronavirus SARS-COV identified in China in the year 2003 [13] and MERS: The coronavirus MERS-COV, first identified in Saudi Arabia in the year 2012 [14].

The current novel Coronavirus (COVID-19) was first found through a group of Chinese people who tested positive for pneumonia [7]. This was at the end of year 2019 in Wuhan city. The disease then spread to their family members and the surrounding people including their health care staff. The contagious nature of this disease resulted in its spread to other 213 countries over few months [6]. 

The coronaviruses circulate in a range of animals. This virus can “spill over” meaning they can jump from animal to human probably due to a range of factors such as mutation or increased contact between human and animals [15]; for example the MERS-COV came from camels [13] and the SARS_COV from civet cats [12]. The animal reservoir of coronavirus (2019-nCoV) is not known yet. 

In general, respiratory viruses are usually transmitted through droplets from an infected person’s cough or sneeze or by touching a surface that has been contaminated with the virus [16]. People at most risk of the coronavirus infection are those who work at an animal market, health care workers treating coronavirus patients, as well as family members caring for infected coronavirus members [17]. The most common symptoms of this coronavirus (2019-nCoV) are fever, tiredness, respiratory symptoms such as cough, sore throat, and shortness of breath, and rare intestinal symptoms such as diarrhoea [18,19]. 

Unluckily, this COVID-19 virus has some features that contributed to its global spread at a relatively short time. The virus has spread all over Asia and reached the United States (Snohomish County, Washington) on 19 January [20] and in Germany on 24 January [21] through different routes from China. Facts about this virus are still new, and what we know about this may change in the future. 

Zhao et al. verified that the initial growth phase of the coronavirus in China was the exponential growth [22]. They used the Serial Intervals (SI) of infection caused by MERS and SARS as approximations for the SI of the coronavirus and estimated the *R*_0_ [22]. Iwato et al. conducted simulations using the SEIR model to assess the impact of secondary outbreaks outside China, assuming that one infected patient travelled to an outside community [23]. While applying the SEIR compartmental model, Kuniya T. predicted the epidemic peak for the coronavirus in Japan using real data from January to February 2020 [24]. Al Qaness et al. developed a novel forecasting model that forecasted and estimated the COVID-19 cases for the upcoming ten days using the Adaptive Neuro-Fuzzy Inference System (ANFIS), which uses the enhanced Flower Pollination Algorithm (FPA) and the Salp Swarm Algorithm (SSA) [25]. Roosa et al., in their study, generated forecasts based on two popular models used previously for forecasting infectious diseases outbreaks, i.e., Richards growth model, and a sub-epidemic wave model [26]. Jung et al. modeled the epidemic growth using two methods, Scenario-1, from a single case recorded on 8 December 2019, and Scenario-2, using the growth rate fitted along with the other parameters based on data from 20 exported cases reported by 24 January 2020 [27]. 

The current study aims to assess the trajectory of the recent pandemic due to the COVID-19 outbreak utilizing a new splitting methodology of the selected countries into two groups and developing regression-based and SIR-based statistical models and tools that depict the actual recorded data of COVID-19. Different modeling techniques potentially provide different prediction results. We considered two modeling techniques that adopt two different prediction approaches to show that although the modeling techniques exhibit different detailed results, they lead to the same general conclusions. This study covers the entire world with emphasis on extreme cases based on the disease containment. Such models will be useful in projecting the COVID-19 trajectory to estimate the daily infection and death rates of the world and selected countries in advance. In addition, this study introduces new factors to be used directly to compare the countries’ responses towards COVID-19. This will help the authorities take the necessary measures and proper action plans to minimize the COVID-19 impact ahead of time. Furthermore, the article also covers some novel potential strategies to contain the virus spread at healthcare settings.

## 2. Materials and Methods 

Actual data (country wide population number, number of infected cases in a country, number of deaths in a country, number of new cases) for the entire world and a few selected countries were obtained from the Worldometer website [6]. According to the website, the data mentioned have been collected from the countries’ health ministry, government institutions, or government authorities’ social media accounts [6]. The data were recorded daily where the day was reset after midnight GMT+0. All the countries recorded new cases for the current day while in progress except China who displayed the previous day cases. Certain countries were chosen to conduct this study based on their outbreak containment and responses. Then, a new splitting methodology was used in the current study to divide the selected countries into two groups (a) Group A: That succeeded in containing the COVID-19 outbreak, where its behavior was split into 3–4 subperiods and (b) Group B: That failed and is still struggling in containing the outbreak, where its behavior was split into two subperiods. Logistic and exponential growth statistical models were used to fit the actual model into regression equations utilizing the excel solver software. In addition, the Susceptible–Infected–Recovered (SIR) Model [28] was applied, which is a compartmental model that has been widely utilized in the literature to predict the spread of infectious diseases. In this model, the Population (*N*) is categorized into three compartments including Susceptible (*S*), Infected (*I*), and Recovered (*R*). The model assumes that the population and both infection and removal rates are constant during the whole epidemic period. In addition, it assumes that the population is well-mixed. During the epidemic period, susceptible cases become infected with a rate β and infected cases become recovered with a rate γ. The rate of change for the three compartments is estimated by the following three differential equations:

(1) $$\frac{dS}{dt}=-\frac{\beta }{N}IS$$

(2) $$\frac{dI}{dt}=\frac{\beta }{N}IS-\gamma I$$

(3) $$\frac{dR}{dt}=\gamma I$$

This model is considered because it requires simple data that is available for the public. Furthermore, its implementation is provided as an open source code. To obtain COVID-19 prediction results for the selected countries, we applied an already existing MATLAB SIR modeling tool [29,30]. The tool takes the daily new infection cases as an input and optimizes the model parameters by minimizing the difference between the actual and estimated number of cases and it considers the possibility of having multiple sub-waves. The number of daily new infection cases were collected from a publicly available repository [31] on 11 July 2020. In this study, an assumption was made that all the data reported by the selected countries were accurate and up-to-date. It is important to note that the results of the prediction models might be inaccurate due to the fact that such models do not consider some affecting factors such as the containment strategies and other related governmental interventions. The considered pandemic prediction models assume that all such related factors remain the same. The prediction results are useful to assess whether more strict interventions must be applied to reduce the estimated number of infections and deaths and relieve the healthcare system.

## 3. Results and Discussion

### 3.1. Actual Data of New Cases

**Figure 1:**
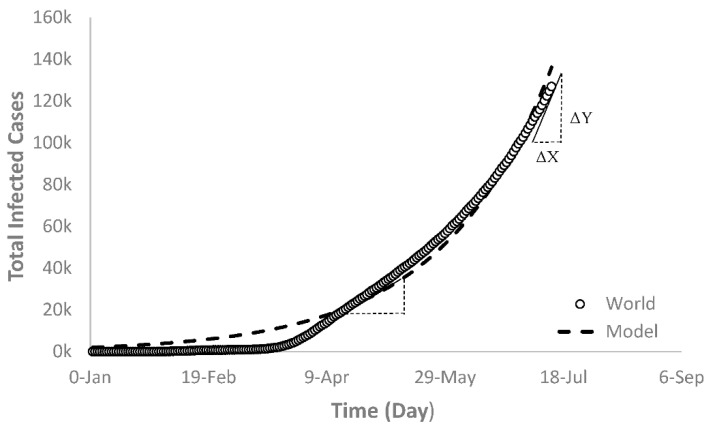
World total infected novel coronavirus (COVID-19) cases.

Figure 1 was generated to show the recorded daily total infected cases of the world over a certain timeframe (22 January 2020 until present), which shows that the COVID-19 disease started to increase slowly until about 11 March and then accelerated at a faster speed afterwards. This means that the infected number of cases after 11 March are significantly higher than the numbers before this date. To illustrate this further, the change in new infected cases for the first 49 days (from 22 January until 10 March) is between 532 to 115,597 cases, whereas the change for a similar period of time (10 March until 26 April)) varied between 115,579 to 2,837,407 cases, which is a 24-fold increase in the new infected cases than that of the first interval. Mathematically, this means that the change in the Y over time (ΔY/ΔX) experienced a relatively drastic jump as opposed to the first interval. In other words, the slope of the second interval is steeper than the first interval (Figure 1), which is also called the tangible line of the graph. This clearly means that the infection is spreading through the world in a faster change rate over a short timeframe. 

**Figure 2:**
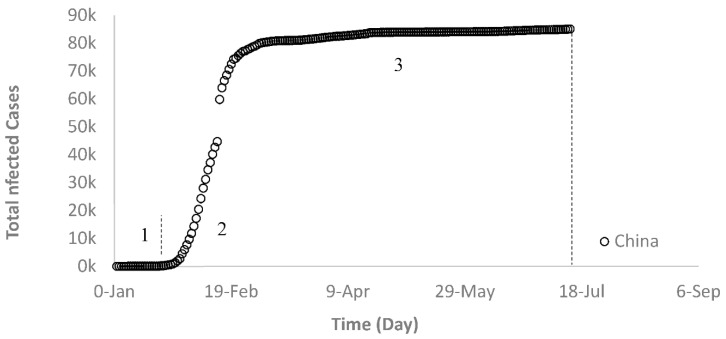
Growth trajectory of COVID-19 cases in China (Group A).

Looking at the selected countries based on containment rates, the countries were classified into two groups: (a) Countries that contained (had controlled) the COVID-19 disease, and (b) those that failed to do so (uncontrolled), as follows (Table 2):

China is taken as an example for Group A where after the outbreak China managed to control the spread of the COVID-19 virus, as shown in Figure 2. China is the origin of COVID-19 where the first cases were recorded in Wuhan city on 22 January 2020. This virus started to spread in China with a short-term (interval 1 in Figure 2) slow rate of infection for about 5 days (22 to 29 January) followed by a faster infection rate in the second interval that lasted for 17 days (26 January to 11 February). Then, the total infected cases continued to increase with a slow rate until it reached the maximum and levelled off thereafter (interval 3). China went through three intervals where interval 1 and 2 represent the breakout stage, and interval 3 the control stage. The slopes for intervals 1 and 2 increased from small to significant and then decreased until they became negligible in interval 3 (Figure 2).

**Figure 3:**
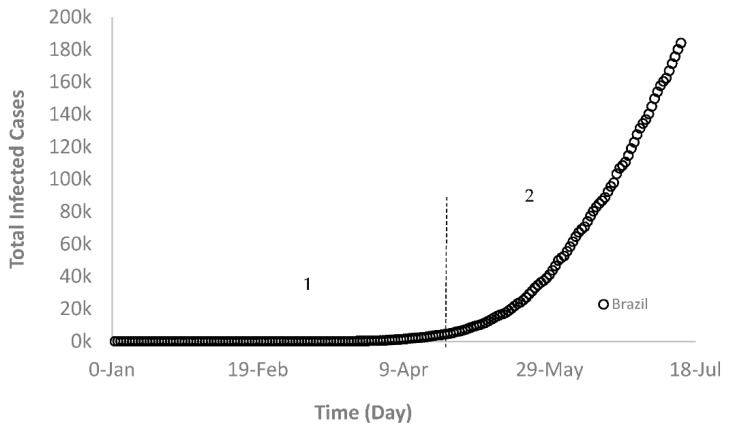
Growth trajectory of COVID-19 cases in Brazil (Group B).

An example of Group B is Brazil, where the COVID-19 spreading rate went through two distinct intervals. Interval 1 where the slope of the curve was negligible indicating small increases in the new cases (3 March to 1 April) and interval 2 represented by a sudden upward shift in the graph as a result of a massive increase in the newly infected cases each day from 20 April onwards (Figure 3). The daily increase rate for interval 1 was from 0 to 5717 new cases, whereas the same rate for interval 2 was from 6836 to 1,839,850, which is significantly higher than that of interval 1 with a notable upward trend. Again, this is due to the drastic change in daily new cases as represented by the changing direction of the slope from a small slope to a steeper one. 

**Figure 4:**
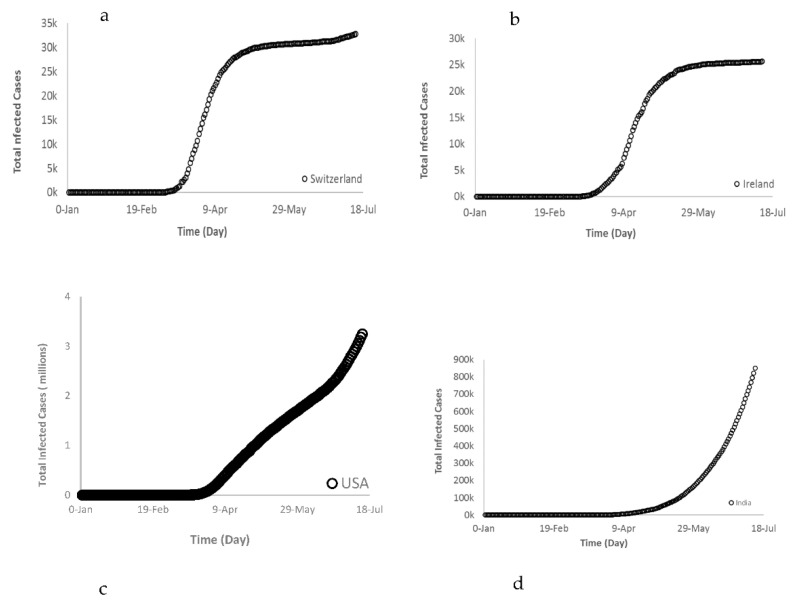
(**a**) Switzerland and (**b**) Ireland as examples of Group A, and (**c**) USA and (**d**) India as examples of Group B.

A set of figures were developed to show the selected countries that exhibited a similar behavior as Groups A and B (Figure 4). 

### 3.2. Reported Deaths

#### 3.2.1. Total Deaths

**Figure 5:**
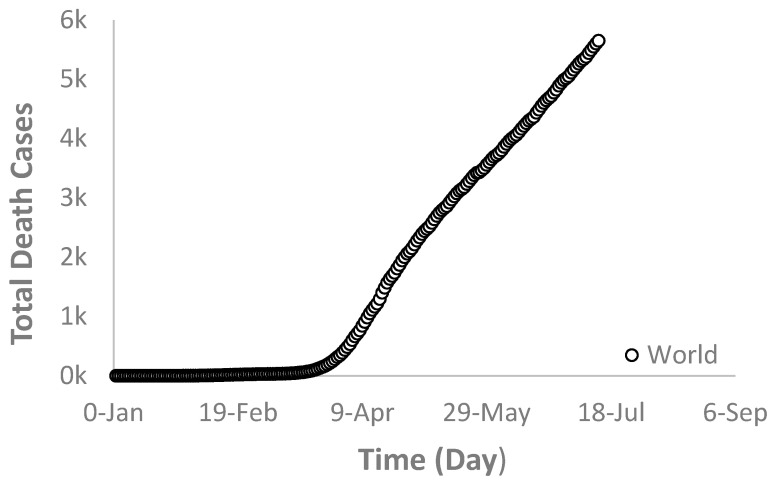
The world deaths due to the novel COVID-19 infections.

**Figure 6:**
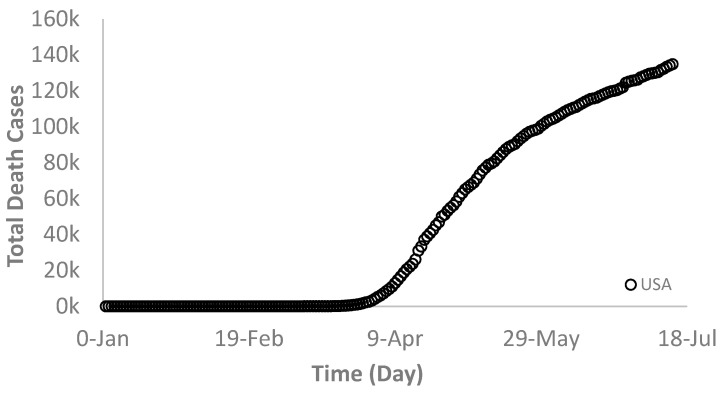
Total deaths due to the novel COVID-19 infections in the USA.

**Figure 7:**
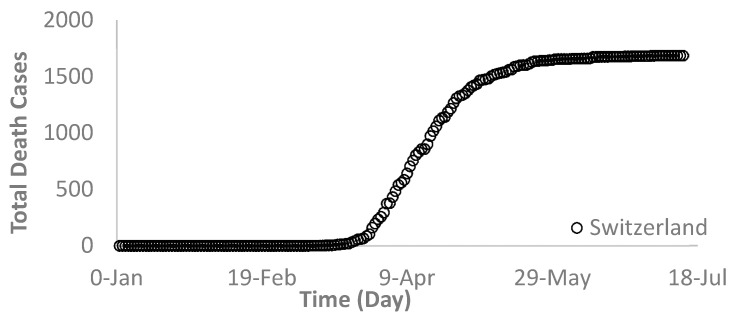
Total deaths due to the novel COVID-19 infections in Switzerland.

Generally, the death rate is proportional to the infection rate but at a smaller scale that varies from country to country, depending on many factors such as population, awareness, health care system, hospital building capacity, demographics, and location. As shown in Figure 5, Figure 6 and Figure 7, the death rates for the world and each selected country depict a similar trend as that of the infection rate. For example, Figure 5 and Figure 6 show the world and the USA death rates, respectively where they are still increasing continuously without reaching a maximum value. In Switzerland, however, the death rate exhibited a similar trend as its infection rate behavior (Figure 7).

**Figure 8:**
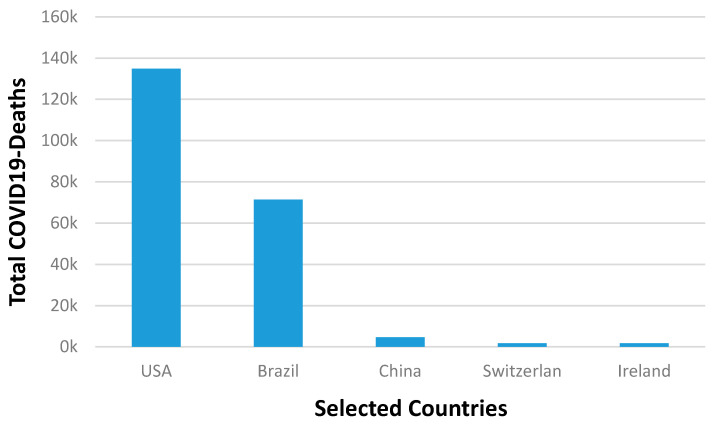
Total deaths due to COVID-19 infections in the selected countries of the world.

Figure 8 is plotted to show the total deaths of the selected countries so far, which shows that USA recorded the highest total deaths due to the COVID-19 infection. However, it would not be accurate to use such data to directly compare the death rates between the countries. Therefore, the next section is dedicated to calculate the death percentage for the selected countries and the world. 

#### 3.2.2. Death Percentage 

A simple Equation (4) is used to calculate the percentage of the deaths for each selected country for the sake of direct comparison. 

Since Population (*P*) is proportional to the number of Infected Cases (*INF*), then:

(4) P α INF

(5) P= A ∗ INF

(6) A =  INFP

where A is the infected cases per capita, which is the infection percentage of the population. Similarly, another factor B that relates the total Deaths (*D*) to the total infected cases is described below:

(7) B= DINF

**Figure 9:**
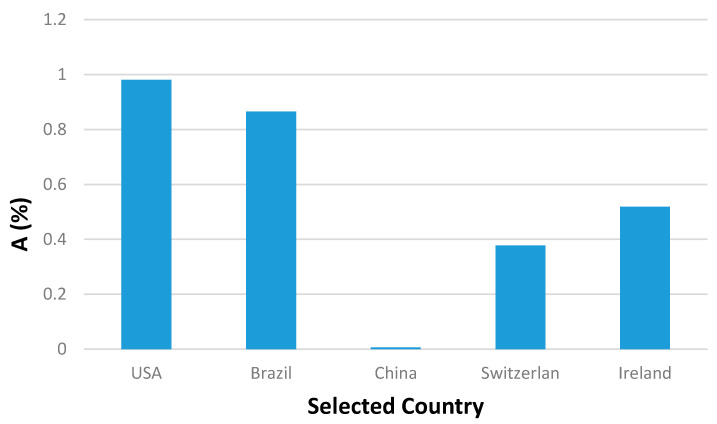
Infected cases per capita, A, for the selected countries of the world.

**Figure 10:**
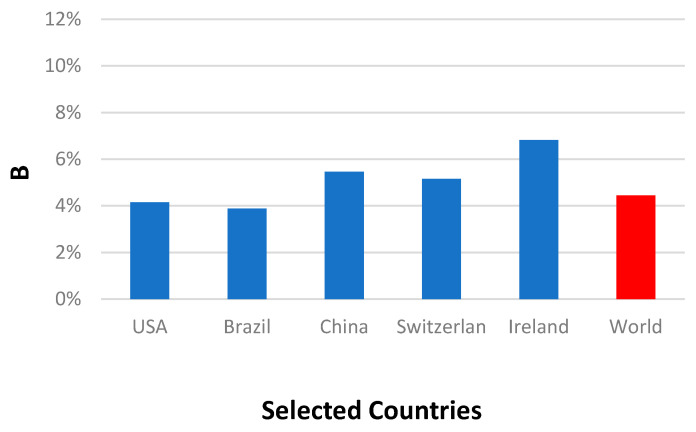
Deaths per infected individual percentage, B, for the selected countries and the world.

Although, China has the largest population in the world, it had the least infection percentage of population (A) compared to the other selected countries (Figure 9). Furthermore, the death percentage (B) of the selected countries up to this date varies between 3.9% (Brazil) to 6.8% (Ireland). The world’s death percentage of 4.45% is within the foregoing values (Figure 10). 

#### 3.2.3. Novel COVID-19 Death Percentages Comparison

**Figure 11:**
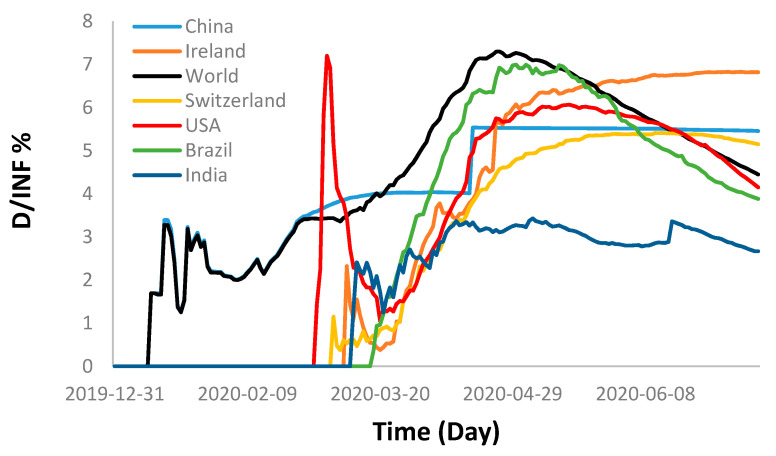
Death percentage rates of some of the selected countries along with the total death cases of the world.

Figure 11 shows the accumulated daily death percentages of the foregoing countries in comparison with the world death percentage, which shows that the world experienced a sharp increase in the total death cases after 7 March until it reached a maximum of 7.3% on 25 April and decreased afterwards until this moment with 4.5%. Group B showed a similar trend to that of the world with slightly less death percentages. This is due to the decline of the daily deaths in comparison with the continuous daily increase in the infected cases. Group A, on the other hand, exhibited a similar behavior at the beginning until it reached a maximum and then levelled off as an indication of containing the outbreak (negligible new infected cases with zero deaths). 

### 3.3. COVID-19 Future Regression-Based Trajectory 

As stated in the previous sections, the trend of COVID-19 outbreak varies between countries at different levels (e.g., death rates, total infected cases, and containment). This section focuses on fitting the actual data of the selected countries into regression-based equations/models that help in understanding the COVID-19 trajectory for a better projection. Once an accurate model is developed (r^2^ > 0.9) for each case, it was used to project the future behavior of COVID-19 to provide potential statistics. This will help in developing proactive action plans and the necessary strategic measures to contain such pandemics in the future too. 

**Figure 12:**
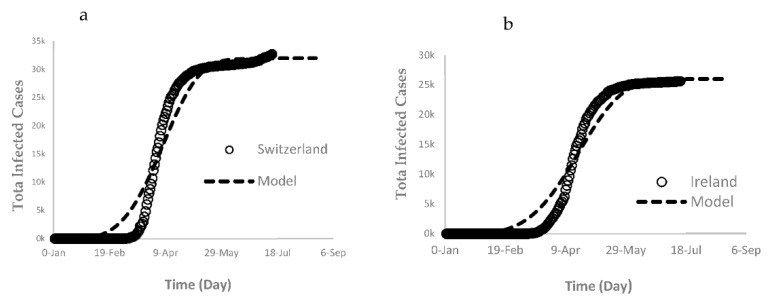
Logistic function regression fit of the total COVID-19 infected cases of Group A countries (**a**) Switzerland and (**b**) Ireland.

Starting with Group A, selected countries such as Switzerland and Ireland, a good regression fit for both countries was obtained from the logistic model with r^2^ > 0.97 (Figure 12) to fit the “S” shaped trend. The fitted model of total COVID-19 cases (*TCOV*) for both countries is expressed by Equation (8):

(8) $$TCOV_{A}\;=M\;\left(1-e^{\left(-k*t^{n}\right)}\right)$$

We have not carried out a sensitivity test in the current study to investigate the effect of each parameter of the logistic model (*M* and *k*) on the infection rate because it is not of our interest at this stage. Given that, they are generally defined as follows, *M* is the amount after growth and *k* is the constant of proportionality (continuous growth). The values of the coefficients for each country are listed in Table 3. 

The trajectory projection of Group A consistently suggests that the pandemic will continue to be contained for all of the Group A countries.

The Group B countries’ trajectory is different from the Group A countries, and therefore a different model was sought to fit such trend. The regression fit that best described the Group B behavior is an exponential growth model with r^2^ ≥ 0.97 as expressed below:

(9) $$TCOV_{B}\;=\;\frac{1}{R}\;\left(e^{\left(\frac{F+t}{R}\right)}\right)$$

where the values of the coefficients for each country in this group are listed in Table 4.

**Figure 13:**
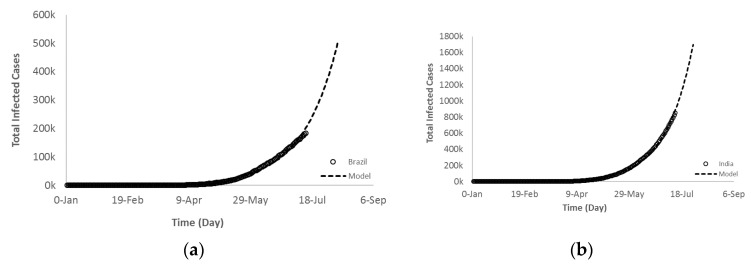
Exponential growth regression fit of the total COVID-19 infected cases of Group B countries (**a**) Brazil and (**b**) India.

Generally, the exponential growth is deceptive because it starts off slowly and after a few days it jumps to enormous numbers. Unfortunately, this is what exactly happened to some of the countries during the COVID-19 pandemic. A set of graphs in Figure 13 shows the current and future trajectory of Brazil and India in Group B. Over the same timeframe, all countries experienced a very slow increase on each day and continued to do so for a few days. This means that the change in *y*-axis was close to zero (slope of the curve). However, after a few doublings the total daily COVID-19 infected cases was increasing with a sharp slope recording higher changes of new cases (ΔY) until it reached enormous numbers. 

If this is not controlled sooner, the total recorded new cases of COVID-19 for these countries will further double claiming more precious lives. For example, the total predicted total COVID-19 infected cases for Brazil, India, and USA by 15 August is expected to be around 6,000,000, 3,259,917, and 5,800,000 with the number of deaths expected to be equal to 200,000, 60,000, and 1,600,000, respectively. Despite the fact that the USA is leading the world in the total recorded infected cases, Brazil will slightly pass the USA by 15 August as predicted by the model. This is due to the fact that the coefficient of the exponential term for Brazil is higher suggesting that the exponential growth stage of Brazil is severer than that of USA. Having said that, the total deaths in the USA will continue to be higher than the rest of the countries. Actual observations proofed that the future actual statistics would be less than the predicted values due to the implementation of physical measures such as social distancing, wearing masks, lock-down, disinfection, and travel restrictions. 

**Figure 14:**
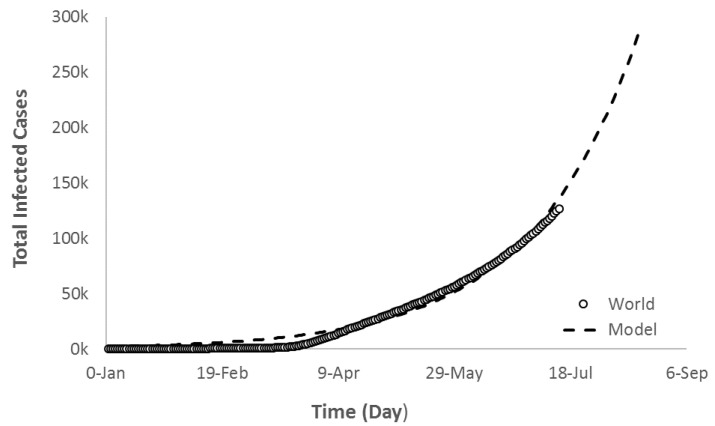
Exponential growth regression fit of the world’s current and future trajectory of COVID-19 total infected cases.

Currently, there are many countries exhibiting a similar trend to Group B that caused the world trajectory to follow an exponential growth model (Figure 14) with r^2^ = 0.99. The world’s total COVID-19 infected cases trajectory was suited more for the exponential growth model (Equation (10)) in the second interval (after 11 March). If the pandemic is not contained, the model (*TCOV_W_*) predicts that the world’s total new infected cases would reach 28,000,000 by 15 August with total deaths equal to 1,500,000.

(10) $$TCOV_{W}\;=\;\frac{1}{46}\;\left(e^{\left(\frac{746+t}{46}\right)}\right)$$

### 3.4. COVID-19 Future SIR-Based Trajectory 

**Figure 15:**
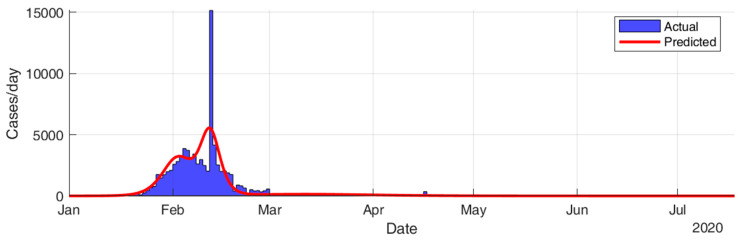
Prediction results for infection cases in China.

**Figure 16:**
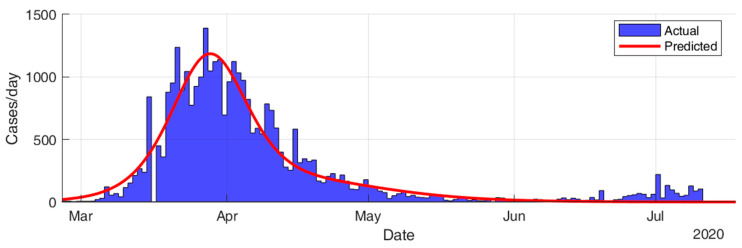
Prediction results for infection cases in Switzerland.

**Figure 17:**
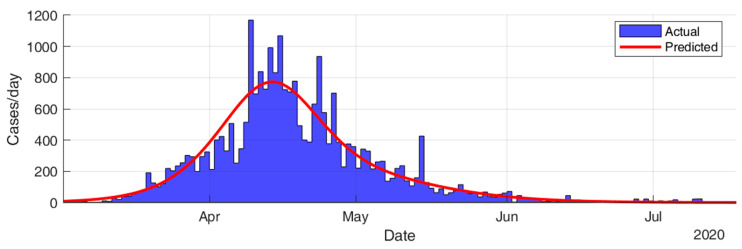
Prediction results for infection cases in Ireland.

**Figure 18:**
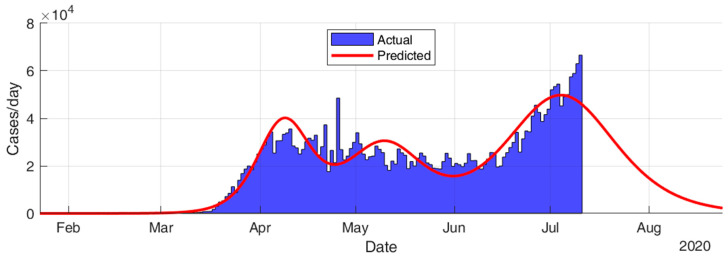
Prediction results for infection cases in USA.

**Figure 19:**
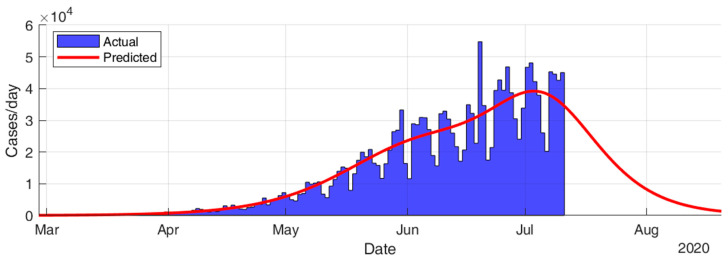
Prediction results for infection cases in Brazil.

**Figure 20:**
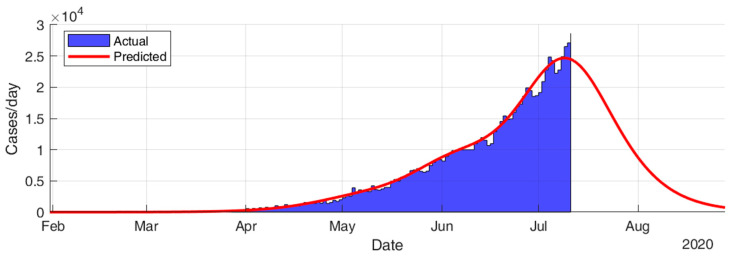
Prediction results for infection cases in India.

This section considers fitting the actual data for the infection cases of the selected countries using SIR modeling and obtaining the prediction results in terms of total number of infection cases and end of pandemic dates. The results given in Table 5 and Figure 15, Figure 16, Figure 17, Figure 18, Figure 19 and Figure 20 show that the countries in Group A are at the end state of the pandemic, which indicates an outbreak control. All these countries already passed the expected 99% end of pandemic dates. The prediction curves for the countries in Group B show that they have just passed the inflection point of the curve and started the deacceleration phase. This indicates that the outbreak is still uncontrolled, and it is expected that these countries reach the outbreak control sometime in August. The prediction curve shows that some of the Group B countries, especially USA, went through several sub-waves of the disease spread. 

### 3.5. Local COVID-19 Study

**Figure 21:**
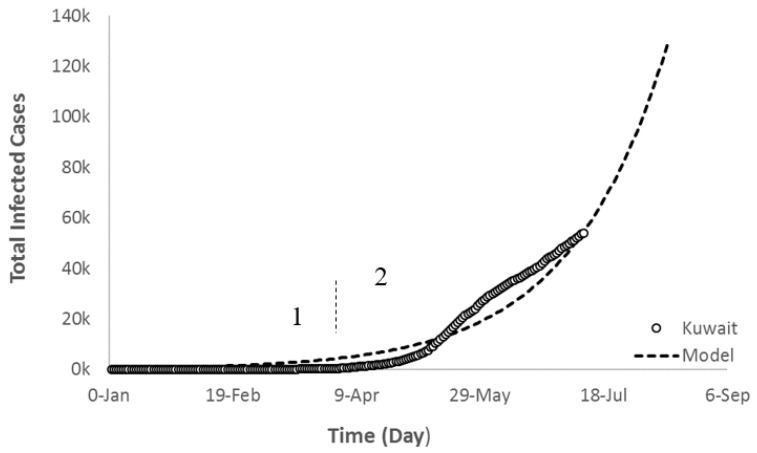
Exponential growth regression fit of Kuwait’s current and future trajectory of COVID-19 total infected cases.

Applying the previous studies on local data for the state of Kuwait resulted in the trajectory depicted in Figure 21. The actual data was fitted to an exponential growth model with an r^2^ > 0.96 as expressed in Equation (5). One can clearly visualize that the slope of the curve has changed since the end of April indicating the start of the exponential growth second stage/interval (2) as those in Group B and the world. This suggests that the state of Kuwait has done a commendable job in reacting right away with this COVID-19 outbreak and took all the necessary measures to control it, which resulted in delaying stage 2 as much as possible. With the current trend, the model predicted that the total infected cases in the state of Kuwait by 15 August would be about 120,000.

(11) $$TCOV\;=\;\;\frac{1}{39}\;\left(e^{\left(\frac{383+t}{39}\right)}\right)$$

**Figure 22:**
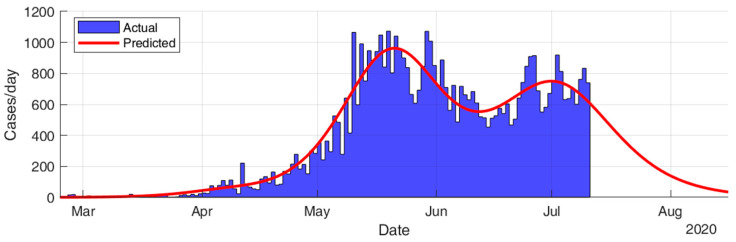
Prediction results for infection cases in Kuwait.

As shown in Figure 22, the results for SIR-modeling for the infection cases in Kuwait show that Kuwait went through two main sub-waves of the disease spread and passed the inflection point of the second sub-wave by the beginning of July. The model predicts the pandemic to end 97% by 29 July 2020 and 99% by 10 August 2020. The total infected cases are predicted to be 62,757, which might be more realistic than the corresponding result of the regression model.

## 4. Potential Prevention/Containment Strategies

The main pathways of COVID-19 spread are through respiratory droplets either coughing, speaking or sneezing, body fluid contact, or touching contaminated surfaces [32]. The prevention/containment of the virus at healthcare settings is more important because it will not only ensure the safety of healthcare workers but will prevent the transmission and spread of the virus. It has also been reported that conventional face masks and ordinary clothing do not provide 100% protection [33]. Therefore, development of new strategies to prevent virus transmission through common pathways is critical. Below we describe current and potential strategies of prevention.

### 4.1. Development of Biodegradable Antiviral Masks

The virus filtering capability of masks depends on the design and materials they are made up of and on the size of the particulates. The current masks have limited ability to protect against aerosol and smaller droplets with surgical and N95 masks having the best protection. Therefore, development of masks with antiviral capabilities can substantially reduce transmission of the virus. Furthermore, current masks from fossil fuel based polymeric materials do not degrade and will create a potential pollution threat to the environment. Therefore, biodegradable antimicrobial masks could be a great potential option.

### 4.2. Antiviral Nano-Coatings of Surfaces

The contaminated surfaces are another contributor to the spread and COVID-19 is reported to remain present on surfaces for several hours to days [34]. The current surface cleaning and disinfection methods are not highly effective where a single wiping of the surfaces becomes dry within 3 min and recovery of the bacteria and viruses is reportedly high [35]. Therefore, efforts should be made to new preventive measures for surface decontaminations. One such method is nano-coating, which can enhance the effectiveness up to several folds compared to current technologies. The emerging self-cleaning nano-coatings have a great future potential to prevent surfaces against such microbial threats.

## 5. Conclusions and Recommendations

**Figure 23:**
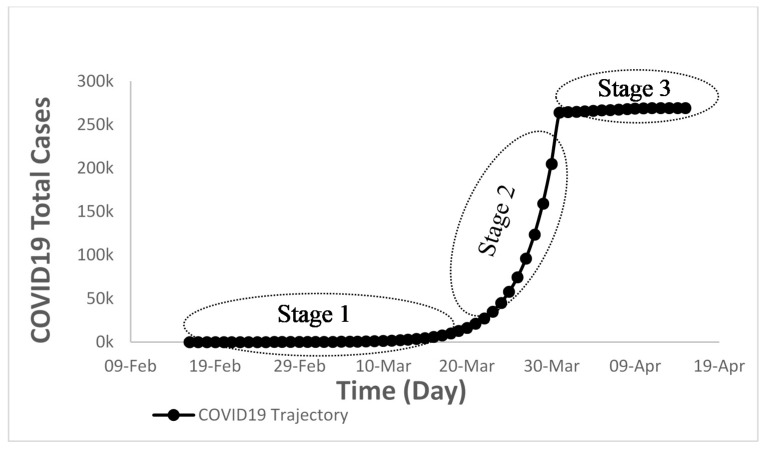
Three critical stages that novel COVID-19 is expected to go through.

Other countries should learn from those who preceded them in the present COVID-19 pandemic. In general, most of the developed countries went through a tough time in dealing with this outbreak. The trajectory of the COVID-19 pandemic, for some countries, went through three critical stages depicting a logistic behavior, as shown in Figure 23. The deceptive stage (1) from the start of the outbreak that varied from one week (in China) to about a month (in USA). Then, it was followed by an uncontrolled exponential increase stage that lasted in some countries for about 20 days (in China, Switzerland, and Ireland) and the rest of the countries unfortunately still experiencing it (USA, Brazil, Spain, and India). The third stage (containment stage) applies to the countries in Group A that controlled the outbreak. 

The maximum impact of this COVID-19 pandemic occurred during the uncontrolled stage (2), which mainly depended on the deceptive stage (1). The shorter the deceptive stage, the shorter the controlling stage and hence the less damage occurred, and vice versa. That is why the countries performance of Group A was better than that of Group B because they took serious measures right from the beginning of the COVID-19 infections (short deceptive stage 1) that resulted in a shorter and less steep uncontrolled stage followed by a controlled stage.

Amongst the Group B countries and based on regression modeling, Brazil could potentially lead the world in the total infected cases in the next few weeks if the circumstances remain the same. However, the SIR-modeling results predict that the USA will continue leading the world in terms of the total infected cases. The state of Kuwait COVID-19 trajectory is similar to that of Group B (uncontrolled). Both regression and SIR-modeling results lead to the same general conclusion that countries in Group A reached the controlling stage, whereas countries in Group B are still far away from reaching this stage. 

**Figure 24:**
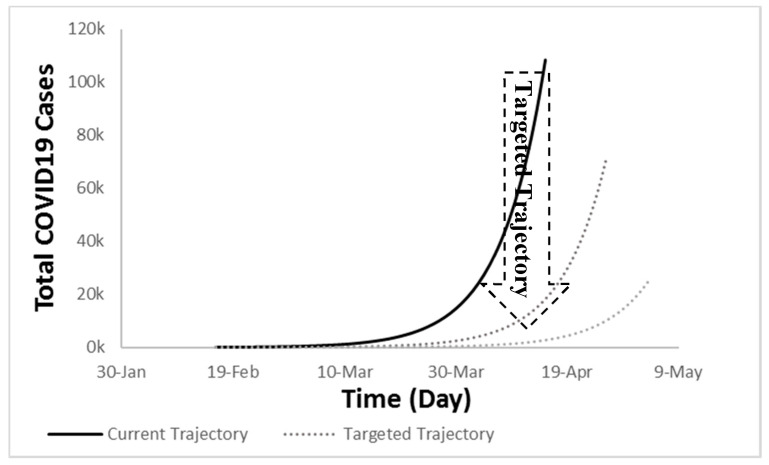
Targeted trajectory of COVID-19 as opposed to the current one.

What needs to be done is to limit the spread of the disease as much as possible. This results in delaying the second stage, and the sharp increase would happen over a longer period instead of on a daily basis that results in reducing the slope of the curve to be less steep (Figure 24). This will spread the new cases over a longer period enabling the health care system to accommodate the existing patients instead of having enormous new cases in a short timeframe. This can be achieved by taking the right measures at the government and individual levels, such as quarantine, personal hygiene, lock-down, curfew, etc. This is what is happening now in some of the countries. 

All the selected countries in both groups recorded lower death percentages than that of the world during the outbreak timeframe. The exponent value (b) of the exponential growth model determines the severity (slope) of the COVID-19 trajectory. That is why the USA is leading the world in the total infected new cases now and Brazil may surpass the USA in the next few weeks if the severity of the exponential remains the same.

Since the model did not consider effects such as containment strategies and other related governmental interventions taken by countries, this limitation could cause slight inaccuracies in the results of the prediction models.

## Figures and Tables

**Table 1:** Historic and recent epidemics/pandemics [3,5].

What	When	Where	Deaths
Black Death	1347–1351	Europe	50,000,000
HIV	1980	Global	39,000,000
Spanish Flu	1918–1920	Global	20,000,000
Asian Flu	1957–1961	Global	2,000,000
Seventh cholera pandemic	1961	Global	570,000
Swine Flu	2009	Global	284,000
Ebola	2014	West Africa	4877
Measles	2011	Congo	4555
SARS	2002–2003	Global	774

**Table 2:** Covid-19 infection cases and deaths reported.

Group A: Controlled			Croup B: Uncontrolled		
Country	Total Infected	Total Deaths	Country	Total Infected	Total Deaths
China	85,071	4641	USA	3,247,684	134,814
Switzerland	32,713	1685	Brazil	1,839,850	71,469
Ireland	25,611	1746	India	849,553	22,674

**Table 3:** Values of coefficients *M*, *k*, and *n* for Switzerland and Ireland.

	Coefficient	*M*	*k*	*n*	r^2^
Country					
Switzerland		31,962.5	5.0 × 10^−9^	4.1	0.99
Ireland		26,000	3.7 × 10^−9^	4.1	0.97

**Table 4:** Values for coefficients F and R for Brazil, India, and USA.

Country	*F*	*R*	r^2^
Brazil	316	29	0.99
India	247	26	0.997
USA	1300	78	0.97

**Table 5:** Susceptible–infected–recovered (SIR)-modeling prediction results.

Group	Country	Expected Number Of Cases	Expected 97% End of Pandemic	Expected 99% End of Pandemic
A	China	84,294	21-March-20	9-April-20
	Switzerland	31,376	11-May-20	25-May-20
	Ireland	25,486	25-May-20	7-June-20
B	USA	4,007,934	6-August-20	18-August-20
	Brazil	2,339,875	3-August-20	14-August-20
	India	1,305,506	10-August-20	22-August-20
